# Antimicrobial-Resistant Infections after Turkey/Syria Earthquakes, 2023

**DOI:** 10.3201/eid2906.230316

**Published:** 2023-06

**Authors:** Anthony Rizk, Antoine Abou Fayad, Louis-Patrick Haraoui

**Affiliations:** Geneva Graduate Institute, Geneva, Switzerland (A. Rizk);; American University of Beirut, Beirut, Lebanon (A. Abou Fayad);; Université de Sherbrooke, Sherbrooke, Québec, Canada (L.-P. Haraoui)

**Keywords:** Antimicrobial resistance, AMR, earthquakes, pathogens, bacteria, Turkey, Syria

## Abstract

Increased rates of multidrug-resistant microbes have been reported after earthquakes. After the 2023 earthquakes in Turkey and Syria, the number of associated highly drug-resistant pathogens and nosocomial transmission will probably surge in hospitals treating injured patients. It is not too late to act to prevent antimicrobial-resistant infections from compounding these tragedies.

The 2023 earthquakes that affected Turkey and Syria, with Kahramanmaraş Province in Turkey at their epicenter, measured 7.8 and 7.5 on the Richter scale. The effects were devastating, making these the strongest earthquakes in Turkey since 1939. Combined with their multiple aftershocks, the earthquakes caused >50,000 deaths and severely damaged or collapsed >170,000 buildings (https://www.aljazeera.com/news/2023/2/25/death-toll-climbs-above-50000-after-turkey-syria-earthquakes). In their wake, the earthquakes left a growing humanitarian crisis. If previous experiences are any indication, we can also expect hospitals caring for the injured and wounded to struggle with highly antimicrobial-resistant infections, many of which will lead to excess illnesses and deaths.

Multidrug resistant microbes have often been reported after earthquakes and other natural disasters. Medical literature on earthquake-associated injuries, going as far back as the Marmara, Turkey, earthquake of 1999 (*1*), have consistently shown highly resistant microbial strains emerging in hospital settings and causing hospital-acquired infections in trauma patients. Antimicrobial-resistant *Acinetobacter baumannii* has been identified in disproportionately high rates from infections associated with large-scale earthquakes in Southeast Asia in 2004; northern Pakistan in 2005; Wenchuan, China, in 2008; central Italy in 2009; and Haiti in 2010 (Appendix).

Although earthquake-associated pathogens detected in hospital settings have been consistently multidrug resistant, their resistance profiles have varied. The etiology of antimicrobial resistance from earthquakes remains uncertain. After the Marmara earthquake of 1999, associated *A. baumannii* and *Pseudomonas aeruginosa* infections were mainly resistant to carbapenems, which had been preemptively administered in large numbers to patients before hospitalization (*1*). After the 2004 Southeast Asia earthquake, patients with multiple wounds admitted to the intensive care unit of the Cologne-Merheim Medical Center in Germany were reportedly contaminated with multidrug-resistant *A. baumannii*, extended-spectrum β-lactamase *Escherichia coli*, and methicillin-resistant *Staphylococcus aureus* (*2*). After the 2005 northern Pakistan earthquake, Kiani et al. reported emergence of multiply drug-resistant *Acinetobacter* spp., *Enterobacter* spp., and other gram-negative organisms, susceptible to amikacin only. Hospitals were overwhelmed with staff and antimicrobial drug shortages, and wounds were often treated empirically because of limited laboratory capacities (*3*). After the 2008 earthquake in Wenchuan, China, the predominant pathogen causing infections was *A. baumannii*; 1 hospital reported that >65% of *Acinetobacter* isolates were resistant to a wide range of antimicrobial drugs, except imipenem, and 24.6% of isolates were pan–drug resistant (*4*).

Since the 2023 Turkey/Syria earthquakes, multidrug resistance has not yet been reported. However, on the basis of the epidemiology of infections in earthquake-stricken regions, we expect hospitals in the region to struggle with containing nosocomial transmission of highly resistant pathogens that may increase deaths among hospitalized patients. Also of concern is the re-emergence of other infectious diseases, such as cholera, known to be precipitated by disaster settings (*5*). The Turkey/Syria earthquakes come on the heels of multiple crises in the region, many of which have been shown to influence the rising rates of multidrug resistance, as in the wake of the Iraq and Syrian conflicts (*6*). Rising rates of antimicrobial resistance are further compounded by the regionwide travel of wounded patients for treatment as healthcare infrastructure in Iraq and Syria collapsed (*7*). Medicine shortages, especially resulting from sanctions in Syria and Iraq and following the 2019 financial collapse in Lebanon, further compound selective pressures on microbes to develop specific resistance profiles.

Hospitals in and outside the region treating injuries from the Turkey/Syria earthquakes will probably observe a surge in highly resistant pathogens and nosocomial transmission. In addition to providing immediate care for the injured and displaced, relief efforts should therefore anticipate a probable increase in antimicrobial-resistant infections for which therapeutic options may be limited. Detecting and responding to this superimposed crisis urgently requires support and expansion of existing microbiology laboratory capacity. Recent initiatives, such as the deployment of telemicrobiology in northern Syria, are examples of how to rapidly assist local laboratory teams in settings of social disruption (*8*). Training healthcare workers on surgical techniques and wound care adapted to deal with traumatic injuries have also been shown to enhance wound healing and to limit complications such as chronic and recurrent skin and soft tissue infections, which are risk factors for antimicrobial resistance (*9*). Early-stage detection and aggressive infection-control practices (e.g., active surveillance, contact isolation, sampling of healthcare workers and hospital environments, and antimicrobial stewardship) during and after disasters play key roles in preventing resistant strains from becoming endemic to healthcare facilities (*10*). Healthcare facilities may need to consider patient decolonization through chlorhexidine bathing to forestall colonization by antimicrobial-resistant *Acinetobacter* strains (*10*). Communities affected by the recent earthquakes will probably experience their effects for months to come. It is not too late to act to prevent further complications from these natural disasters, such as antimicrobial-resistant infections, from compounding ongoing human tragedies.

Appendix Additional information with regard to antimicrobial-resistant infections after Turkey/Syria earthquakes, 2023.
