# Supplementary material for: Antimicrobial-Resistant Infections after Turkey/Syria Earthquakes, 2023
Source: Emerg Infect Dis. 2023 Jun;29(6):1273–5. doi: 10.3201/eid2906.230316 (PMC10202876; doi:10.3201/eid2906.230316)
Supplement: Appendix — Additional information with regard to antimicrobial-resistant infections after Turkey/Syria earthquakes, 2023. [file 23-0316-Techapp-s1.pdf]

Article DOI: https://doi.org/10.3201/eid29065.230316

*EID cannot ensure accessibility for supplementary materials supplied by authors. Readers who have difficulty accessing supplementary content should contact the authors for assistance.*

# Antimicrobial-Resistant Infections after Turkey/Syria Earthquakes, 2023

## Appendix

**Table.** Characteristics of earthquake-associated infections with antibiotic-resistant pathogens

| Earthquake location, date | Severity | Casualties | Key contaminants of earthquake-associated wounds |
|---|---|---|---|
| Marmara, Turkey, 1999 | 7.4 magnitude, affecting an area of 200 km × 40 km | 17,480 deaths, 43,953 injured | *Acinetobacter* spp. and *Pseudomonas aeruginosa* resistant to carbapenems and sensitive to quinolones; methicillin-resistant *Staphylococcus aureus* (*1*) |
| Southeast Asia, 2004 | 9.1 magnitude, triggering a massive tsunami | 310,000 deaths, millions destitute | MDR *Acinetobacter* spp.; ESBL *Escherichia coli*; methicillin-resistant *Staphylococcus aureus* (MRSA); and *Aeromonas hydrophilia*, *Pseudomonas* spp., and *Candida albicans* (*2*). |
| Northern Pakistan, 2005 | 7.6 magnitude, with 140 aftershocks | >82,000 deaths, 3.3 million injured | MDR *Pseudomonas* spp., *Enterobacter* spp., and *Acinetobacter* spp. only susceptible to amikacin (*3*). |
| Wenchuan, China, 2008 | 8.0 maginitude | >69,000 deaths, 45.5 million destitute | MDR *Acinetobacter baumannii*, *Escherichia coli, Pseudomonas aeruginosa* and *Staphylococcus aureus* (*4*,*5*). |
| Central Italy, 2009 | 5.9 magnitude | 308 deaths, >1,000 injured | Extensively drug-resistant *Acinetobacter baumannii* belonging to *A. baumannii* sequence type 2 with $bla_{OXA-23}$ (*6*) |
| Haiti, 2010 | 7.0 magnitude | >100,000 deaths | Three *A. baumannii* isolates belonging to 2 distinct clones and were identified as ESBL producers and found to be $bla_{CTX-M-15}$-positive. They were resistant to penicillins, broad-spectrum cephalosporins and aztreonam but susceptible to carbapenems (*7*). |

### References

1. Öncül O, Keskin O, Acar HV, Küçükardali Y, Evrenkaya R, Atasoyu EM, et al. Hospital-acquired infections following the 1999 Marmara earthquake. J Hosp Infect. 2002;51:47–51. PubMed https://doi.org/10.1053/jhin.2002.1205

2. Maegele M, Gregor S, Steinhausen E, Bouillon B, Heiss MM, Perbix W, et al. The long-distance tertiary air transfer and care of tsunami victims: injury pattern and microbiological and psychological aspects. Crit Care Med. 2005;33:1136–40. PubMed https://doi.org/10.1097/01.CCM.0000163269.42524.50

3. Kiani QH, Amir M, Ghazanfar MA, Iqbal M. Microbiology of wound infections among hospitalised patients following the 2005 Pakistan earthquake. J Hosp Infect. 2009;73:71–8. PubMed https://doi.org/10.1016/j.jhin.2009.06.012

4. Wang Y, Hao P, Lu B, Yu H, Huang W, Hou H, et al. Causes of infection after earthquake, China, 2008. Emerg Infect Dis. 2010;16:974–5. PubMed https://doi.org/10.3201/eid1606.091523

5. Tao C, Kang M, Chen Z, Xie Y, Fan H, Qin L, et al. Microbiologic study of the pathogens isolated from wound culture among Wenchuan earthquake survivors. Diagn Microbiol Infect Dis. 2009;63:268–70. PubMed https://doi.org/10.1016/j.diagmicrobio.2008.11.009

6. Perilli M, Sabatini A, Pontieri E, Celenza G, Segatore B, Bottoni C, et al. OXA-23 carbapenemase in multidrug-resistant *Acinetobacter baumannii* ST2 type: first identification in L'Aquila Hospital (Italy). Microb Drug Resist. 2015;21:97–101. PubMed https://doi.org/10.1089/mdr.2014.0056

7. Potron A, Munoz-Price LS, Nordmann P, Cleary T, Poirel L. Genetic features of CTX-M-15-producing *Acinetobacter baumannii* from Haiti. Antimicrob Agents Chemother. 2011;55:5946–8. PubMed https://doi.org/10.1128/AAC.05124-11
